# Fish consumption in relation to myocardial infarction, stroke and mortality among women and men with type 2 diabetes: A prospective cohort study

**DOI:** 10.1016/j.clnu.2017.01.012

**Published:** 2018-04

**Authors:** Alice Wallin, Nicola Orsini, Nita G. Forouhi, Alicja Wolk

**Affiliations:** a Unit of Nutritional Epidemiology, Institute of Environmental Medicine, Karolinska Institutet, Box 210, 171 77 Stockholm, Sweden; b Medical Research Council Epidemiology Unit, University of Cambridge School of Clinical Medicine, Box 285, Institute of Metabolic Science, Cambridge Biomedical Campus, Cambridge CB2 0QQ, United Kingdom

**Keywords:** Type 2 diabetes, Fish consumption, Prospective cohort study, Myocardial infarction, Stroke, Cardiovascular disease, BMI, body mass index, CHD, coronary heart disease, CI, confidence interval, COSM, Cohort of Swedish Men, CVD, cardiovascular disease, DASH, Dietary Approaches to Stop Hypertension, FFQ, food frequency questionnaire, HR, hazard ratio, ICD, International Classification of Diseases, MI, myocardial infarction, PCB, polychlorinated biphenyl, SMC, Swedish Mammography Cohort

## Abstract

**Background & aims:**

The accumulated evidence supports an inverse association of fish consumption with cardiovascular disease and mortality, but data among patients with type 2 diabetes are sparse. We aimed to assess fish consumption in relation to myocardial infarction (MI), stroke and mortality among individuals with type 2 diabetes.

**Methods:**

Women and men with diagnosed type 2 diabetes (n = 2225; aged 45–84 years) within two population-based cohorts (the Swedish Mammography Cohort and the Cohort of Swedish Men) were followed from 1998 through 2012. Cox proportional hazards models were used to estimate hazard ratios (HRs) with 95% confidence intervals (CIs).

**Results:**

We identified 333 incident MI events, 321 incident stroke events and 771 deaths (154 with coronary heart disease [CHD] as underlying cause) during follow-up of up to 15 years. The multivariable HRs comparing >3 servings/week with ≤3 servings/month were 0.60 (95% CI, 0.39–0.92) for MI and 1.04 (95% CI, 0.66–1.64) for stroke. HRs for total mortality were lowest for moderate fish consumption of 1–<2 servings/week (0.82; 95% CI, 0.64–1.04) and 2–3 servings/week (0.79; 95% CI, 0.61–1.01) compared with ≤3 servings/month. The corresponding HRs for CHD-related mortality were 0.53; 95% CI, 0.32–0.90 and 0.75; 95% CI, 0.45–1.27.

**Conclusions:**

Fish consumption was associated with lower MI incidence among individuals with type 2 diabetes, whereas no association was observed with stroke. Our data further indicated an association with lower mortality, particularly for CHD-related deaths. These findings support the current general advice on regular fish consumption also in the high risk group of type 2 diabetes patients.

## 1 Introduction

The accumulated evidence generally supports an inverse association of fish consumption with cardiovascular disease (CVD) and overall mortality [1], [2], [3], [4]. Agencies and organizations in different countries advise at least two servings of fish per week for cardiovascular health promotion in the general population, especially emphasizing fatty fish rich in omega-3 [5], [6]. Type 2 diabetes is associated with higher CVD incidence as well as with higher mortality rates compared with the general population, and CVD constitute the leading cause of death in this patient group [7]. The excess CVD risk is likely explained both by a higher burden of classic risk factors like elevated LDL cholesterol and blood pressure, and by specific metabolic changes following insulin resistance and hyperglycemia [8]. Dietary counseling is a key component in diabetes management, largely aimed toward prevention of CVD [9]. However, there are gaps in the scientific evidence for dietary recommendations in this group of patients, including sparse data on the association between fish consumption and CVD risk [10], [11], [12] and mortality [10], [13], [14]. Specific recommendations for people with type 2 diabetes follows the recommendations on fish consumption for the general public and are largely based on the same evidence [9], [15]. We therefore aimed to assess consumption of total and specific types of fish in relation to risk of myocardial infarction (MI), stroke and mortality among participants with diagnosed type 2 diabetes in two prospective cohorts of women and men.

## 2 Materials and methods

### 2.1 Study population

This study is based on data from two prospective cohorts, the Swedish Mammography Cohort (SMC) and the Cohort of Swedish Men (COSM) [16]. In 1987–1990, all women born 1914–1948 and living in Uppsala or Västmanland County of central Sweden were invited to participate in the SMC. They received a mailed a questionnaire on diet and other lifestyle factors, which 66,651 women completed (74% response rate). The questionnaire information was updated in the autumn of 1997 when an expanded version was sent to all women still alive. Simultaneously, a parallel cohort of men, the COSM, was initiated. The questionnaire was distributed to all men born 1918–1952 and living in Västmanland or Örebro County. The study population for the present study is derived from the women (n = 39,227; response rate 70%) and men (n = 48,850; response rate 49%) who returned the 1997 questionnaire. We excluded women and men with incomplete/incorrect national identification numbers (n = 448), those who returned blank questionnaires (n = 92), those who died (n = 97), had a cancer diagnosis (excluding non-melanoma skin cancer; n = 4298) or CVD (MI, angina or stroke; n = 7799) before 1 January 1998, those with implausible total energy intake (±3SD from the log-transformed mean; n = 860); those with missing data on all items in the fish intake section (n = 442) and those with missing on frying frequency (n = 3233). Among the remaining 70,808 participants, only those with a diagnosis of type 2 diabetes before baseline (1 January 1998) were included in the present study. Participants with diabetes were identified by linkage to the Swedish National Diabetes Register and the Swedish National Patient Register. The date of diagnosis was defined as first available date in either of the two registers, or derived from retrospectively recorded information on diabetes onset in the National Diabetes Register. The final study cohort of type 2 diabetes patients comprised 2225 women and men. The study has been approved by the Regional Ethical Review Board in Stockholm, Sweden.

### 2.2 Assessment of diet and covariates

Dietary habits were queried with a food frequency questionnaire (FFQ) including 96-items. For each food item, the participants were asked to report their average consumption frequency during the previous year. Eight predefined frequency categories were provided, ranging from less than once per month to three or more times per day. Three items concerned different types of finfish (herring/mackerel, salmon/whitefish/char and cod/saithe/fish fingers) and one item concerned shellfish (shrimp/crayfish etc.). Single missing answers in the section on fish consumption were assumed to mean never/seldom. The responses on the three finfish items were summed using the frequency categories' midpoints to obtain a value for total fish consumption. Monthly consumption of different fried foods, including fish fried in a pan, was queried in a separate questionnaire section without predefined response categories. In a validation study based on 248 men of the same age and from the same study area, the Spearman rank correlation coefficients between the FFQ-based estimates and the mean of 14 24-h recall interviews were 0.65 for macronutrients and 0.62 for micronutrients [17]. In a sample of 129 SMC women, the correlation coefficients between intake estimated from the FFQ and from four 1-week weighted diet records were 0.5 for fatty fish, 0.4 for lean fish, and 0.6 for shellfish [18].

As an estimate of overall quality of the diet, we calculated a Dietary Approaches to Stop Hypertension (DASH) diet component score [19], [20], [21]. This dietary score has previously been inversely associated with type 2 diabetes risk [22], [23] and does not include fish intake. Points are awarded for high intake of fruit, vegetables, nuts and legumes, low-fat dairy and whole grains, and for low intake of sodium, sweetened beverages, and red and processed meats.

From the questionnaire we also obtained data on height, weight, physical activity, education, smoking habits, alcohol consumption, history of high cholesterol and hypertension, family history of MI, and use of aspirin and fish oil supplements.

### 2.3 Ascertainment of myocardial infarction, stroke and total mortality

Participants were followed for first hospitalization or death from MI or stroke as well as for death from any cause during follow-up. Cases were ascertained through record linkage to the Swedish National Patient Register and Causes of Death Register which provide virtually complete national coverage. The International Classification of Diseases (ICD)-10 code I21 for MI and codes I60, I61, I63 and I64 for stroke listed as the primary diagnosis were used. For mortality, we separately assessed deaths with coronary heart disease (CHD) as underlying cause (ICD-10 codes I20–I25).

### 2.4 Statistical analysis

For CVD incidence, each participant contributed person-time from 1 January 1998 until date of first MI or stroke, date of death, or end of follow-up (31 December 2012), whichever occurred first. For mortality, person-time was accumulated until date of death from any cause or end of follow-up. We used Cox proportional hazard models to estimate hazard ratios (HRs) with 95% confidence intervals (CIs) of MI, stroke, total mortality and CHD-related mortality by categories of total fish (≤3 servings/month; 1–<2 servings/week; 2–3 servings/week; >3 servings/week), individual fish and shellfish items (<1 serving/month; 1–3 servings/month; ≥1 serving/week), and monthly fried fish consumption (tertiles). Age was used as the underlying time scale. No violations of the proportional hazards assumption were indicated using the Schoenfeld residual test. In multivariable-adjusted analyses, we adjusted for time since diabetes diagnosis (years, continuous), BMI (kg/m^2^; <20, 20–24.9, 25–29.9, ≥30), physical activity (<20, 20–40, >40 min of walking or bicycling per day), education (primary school, high school, university), cigarette smoking (never, former <10 pack-years or ≥10 pack-years, current < 20 pack-years or ≥20 pack-years), total energy intake (kcal/day; sex-specific quartiles), alcohol (g/day, sex-specific quartiles), history of high cholesterol (yes/no), history of hypertension (yes/no) and DASH diet component score (quartiles). Individual fish and shellfish items were additionally mutually adjusted. Missing data for covariates were treated as separate categories. To test for linear trend, we used the median within each fish consumption category as a continuous variable in the model. Tests for interaction by sex were performed with the likelihood ratio test. In sensitivity analyses, we excluded cases that were identified during the first two years of follow-up. Further, we performed stratified analyses by diabetes duration (less or more than the median of 6 years). To allow for assessment of potential interactions with age, we additionally conducted sensitivity analyses using follow-up time as the time-scale. The statistical analyses were conducted using Stata (version 13; StataCorp LP, College Station, TX).

## 3 Results

During 26,188 person-years of follow-up (mean, 11.8 years), 333 incident MI events and 321 incident stroke events (284 ischemic, 13 hemorrhagic, 24 undefined) were ascertained. Follow-up for mortality was 29,435 person-years (mean, 13.2 years), during which 771 deaths occurred (154 with CHD as underlying cause). The mean (±SD) total fish consumption at baseline was 2.2 (±2.3) servings/week, including 0.8 (±1.1) servings of herring/mackerel, 0.4 (±1.0) servings of salmon/whitefish/char and 1.0 (±1.4) servings of cod/saithe fish fingers. For shellfish and fried fish consumption, the means were 0.4 (±0.6) servings/week and 3.3 (±2.9) times/month, respectively. Table 1 shows age-standardized baseline characteristics by sex and total fish consumption. Participants with higher fish consumption had on average higher total energy intake and more frequent consumption of fried fish. They were also more likely to have a university education, to be in the higher quartiles of the DASH dietary component score and to have had hypertension and high cholesterol. Women who consumed more fish were less likely to currently smoke and more likely to have a family history of MI. Men who consumed more fish had on average higher physical activity and were more likely to be fish oil supplement users.

**Table 1:** Age-standardized characteristics of participants from the Swedish Mammography Cohort (aged 50–84 years) and the Cohort of Swedish Men (aged 45–79 years) with type 2 diabetes at baseline 1998, by sex and total fish consumption.^a^

**Total fish consumption, servings (median)**								
**Characteristics**	Women (n = 912)				Men (n = 1313)			
	**≤3/month** (0.5/week)	**1–<2/week** (1.4/week)	**2–3/week** (2.4/week)	**>3/week** (4.0/week)	**≤3/month** (0.5/week)	**1–<2 /week** (1.4/week)	**2–3/week** (2.4/week)	**>3/week** (3.5/week)
No. of participants	81	330	316	185	151	581	400	181
Age (mean ± SD, years)	66.0 ± 9.4	64.2 ± 8.8	66.0 ± 8.6	66.9 ± 8.6	62.2 ± 8.8	62.5 ± 8.8	62.9 ± 8.5	65.4 ± 8.1
Time since type 2 diabetes diagnosis (mean, years)	8.5	7.7	7.2	7.6	7.3	7.1	8.0	7.4
BMI (mean, kg/m^2^)	28	28	27	28	28	27	28	28
Physical activity (>40 min of walking or bicycling/day, %)	45	34	38	41	24	34	31	40
University education (%)	3	9	11	13	10	13	14	14
Current smokers (%)	24	20	18	16	24	22	22	25
Alcohol (mean, g/day)	2	4	5	6	13	13	13	14
Energy intake (mean, kcal/day)	1500	1610	1750	1910	2300	2430	2560	3000
DASH diet component score^b^								
Quartile 1 (%)	28	17	14	12	50	40	31	25
Quartile 2 (%)	32	22	21	18	26	28	30	26
Quartile 3 (%)	16	31	26	24	16	19	25	25
Quartile 4 (%)	21	30	39	46	5	11	14	24
Fried fish consumption (mean, times/month)	2.2	2.8	3.3	3.8	2.4	3.2	3.8	4.8
History of hypertension (%)	36	40	45	49	39	40	41	41
History of high cholesterol (%)	7	10	12	22	14	14	14	16
Family history of MI (%)	13	16	19	19	12	11	11	12
Aspirin use (%)	38	41	44	41	36	35	31	38
Fish oil supplement use (%)	9	5	7	9	2	4	4	8

a Total fish is the sum of three finfish items: herring/mackerel, salmon/whitefish/char and cod/saithe/fish fingers; all variables except age are standardized to the age distribution of the study cohorts of women and men; data were missing on BMI for 109 participants, physical activity for 183 participants, education for 28 participants, smoking status for 33 participants, alcohol intake for 479 participants and diet score for 23 participants.

b Dietary Approaches to Stop Hypertension score based on intake of fruits, vegetables, nuts and legumes, low-fat dairy, whole grains, sodium, sweetened beverages, and red and processed meats [19].

Tests for interaction by sex were not statistically significant for the associations between total fish and MI (p_interaction_ = 0.09), stroke (p_interaction_ = 0.19), total mortality (p_interaction_ = 0.17) or CHD-related mortality (p_interaction_ = 0.16). Therefore, we present all results for women and men combined. In the multivariable-adjusted models, fish consumption was inversely associated with MI incidence but not with stroke (Table 2). Compared with those who consumed up to three servings/month of total fish, the multivariable HR of MI was 0.60 (95% CI, 0.39–0.92) for those who consumed more than three servings/week. When assessing individual fish and shellfish items in relation to MI incidence, we observed an inverse association with herring/mackerel, whereas no association was observed with salmon/whitefish/char or shellfish. Moderate consumption (1–3 servings/month) of cod/saithe/fish fingers was also inversely associated with MI.

**Table 2:** Hazard ratios of myocardial infarction and stroke, according to categories of total fish, fatty fish, lean fish and shellfish consumption in 2225 women and men with type 2 diabetes, the Cohort of Swedish Men and the Swedish Mammography Cohort 1998–2012.

	Myocardial infarction				P_trend_	Stroke				P_trend_
**Total fish**^a^ servings (median)	**≤3/month** (0.5/week)	**1–<2/week** (1.4/week)	**2–3/week** (2.4/week)	**>3/week** (3.5/week)		**≤3/month** (0.5/week)	**1–<2/week** (1.4/week)	**2–3/week** (2.4/week)	**>3/week** (3.5/week)	
No. of cases	48	130	107	48		31	135	94	61	
Person-years	2569	10,913	8548	4157		2569	10,913	8548	4157	
Age- and sex-adjusted	1.00 (ref)	0.63 (0.45–0.88)	0.65 (0.46–0.92)	0.57 (0.38–0.85)	0.05	1.00 (ref)	0.99 (0.67–1.47)	0.87 (0.58–1.30)	1.05 (0.68–1.62)	0.94
Multivariable model^b^	1.00 (ref)	0.66 (0.47–0.92)	0.67 (0.47–0.96)	0.60 (0.39–0.92)	0.08	1.00 (ref)	1.02 (0.68–1.51)	0.89 (0.58–1.35)	1.04 (0.66–1.64)	0.86
**Herring/mackerel** servings (median)	**<1/month** (0/week)	**1–3/month** (0.5/week)	**≥1/week** (1.5/week)			**<1/month** (0/week)	**1–3/month** (0.5/week)	**≥1/week** (1.5/week)		
No. of cases	77	182	74			65	166	90		
Person-years	4983	14,666	6538			4983	14,666	6538		
Age- and sex-adjusted	1.00 (ref)	0.81 (0.62–1.06)	0.68 (0.49–0.93)		0.03	1.00 (ref)	0.90 (0.68–1.20)	0.95 (0.69–1.32)		0.99
Multivariable model^b^	1.00 (ref)	0.89 (0.67–1.18)	0.70 (0.50–0.98)		0.03	1.00 (ref)	0.87 (0.64–1.18)	0.89 (0.63–1.25)		0.67
**Salmon/whitefish/char** servings (median)	**<1/month** (0/week)	**1–3/month** (0.5/week)	**≥1/week** (1.5/week)			**<1/month** (0/week)	**1–3/month** (0.5/week)	**≥1/week** (1.5/week)		
No. of cases	157	148	28			137	149	35		
Person-years	10,860	12,836	2491			10,860	12,836	2491		
Age- and sex-adjusted	1.00 (ref)	0.87 (0.69–1.09)	0.79 (0.53–1.18)		0.18	1.00 (ref)	1.05 (0.83–1.33)	1.11 (0.77–1.61)		0.55
Multivariable model^b^	1.00 (ref)	0.94 (0.74–1.20)	0.86 (0.57–1.31)		0.46	1.00 (ref)	1.02 (0.80–1.32)	1.05 (0.71–1.55)		0.80
**Cod/saithe/fish fingers** servings (median)	**<1/month** (0/week)	**1–3/month** (0.5/week)	**≥1/week** (1.5/week)			**<1/month** (0/week)	**1–3/month** (0.5/week)	**≥1/week** (1.5/week)		
No. of cases	48	146	139			35	145	141		
Person-years	2590	12,808	10,789			2590	12,808	10,789		
Age- and sex-adjusted	1.00 (ref)	0.62 (0.45–0.86)	0.69 (0.50–0.96)		0.54	1.00 (ref)	0.84 (0.58–1.22)	0.93 (0.64–1.35)		0.69
Multivariable model^b^	1.00 (ref)	0.64 (0.46–0.90)	0.75 (0.53–1.05)		0.89	1.00 (ref)	0.86 (0.58–1.25)	0.94 (0.64–1.39)		0.71
**Shellfish** servings (median)	**<1/month** (0/week)	**1–3/month** (0.5/week)	**≥1/week** (1.5/week)			**<1/month** (0/week)	**1–3/month** (0.5/week)	**≥1/week** (1.5/week)		
No. of cases	139	160	34			113	168	40		
Person-years	8582	14,713	2892			8582	14,713	2892		
Age- and sex-adjusted	1.00 (ref)	0.77 (0.61–0.97)	0.81 (0.56–1.18)		0.17	1.00 (ref)	1.09 (0.85–1.38)	1.26 (0.88–1.81)		0.21
Multivariable model^b^	1.00 (ref)	0.80 (0.62–1.03)	0.85 (0.57–1.28)		0.35	1.00 (ref)	1.13 (0.87–1.47)	1.23 (0.83–1.82)		0.30

a Total fish is the sum of herring/mackerel, salmon/whitefish/char and cod/saithe/fish fingers.

b Adjusted for attained age, sex, time since diabetes diagnosis (years, continuous), BMI (kg/m^2^; <20, 20–24.9, 25–29.9,≥30) , physical activity (<20, 20–40, >40 min of walking or bicycling per day), education (primary school, high school, university), cigarette smoking (never, former <10 pack-years or ≥10 pack-years, current <20 pack-years or ≥20 pack-years), total energy intake (kcal/day; sex-specific quartiles), alcohol (g/day, sex-specific quartiles), history of high cholesterol (yes/no), history of hypertension (yes/no) and DASH diet component score (based on intake of fruits, vegetables, nuts and legumes, low-fat dairy, whole grains, sodium, sweetened beverages, and red and processed meats; quartiles). Individual fish and shellfish items were mutually adjusted.

HRs of total and CHD-related mortality according to fish consumption are presented in Table 3. The HRs for the association with total fish were not statistically significant but indicated inverse point estimates, with the lowest HR among those consuming 2–3 servings/week (HR, 0.79; 95% CI, 0.61–1.01). For CHD-related mortality, 1–<2 servings/week of total fish was associated with lower risk (HR, 0.53; 95% CI, 0.32–0.90). Among individual fish items, only moderate consumption (1–3 servings/month) of cod/saithe/fish fingers was statistically significantly associated with total and CHD-related mortality.

**Table 3:** Hazard ratios of total and coronary heart disease-related mortality, according to categories of total fish, fatty fish, lean fish and shellfish consumption in 2225 women and men with type 2 diabetes, the Cohort of Swedish Men and the Swedish Mammography Cohort 1998–2012.

	Total mortality				P_trend_	Coronary heart disease-related mortality				P_trend_
**Total fish**^a^ servings (median)	**≤3/month** (0.5/week)	**1–<2/week** (1.4/week)	**2–3/week** (2.4/week)	**>3/week** (3.5/week)		**≤3/month** (0.5/week)	**1–<2/week** (1.4/week)	**2–3/week** (2.4/week)	**>3/week** (3.5/week)	
No. of cases	92	292	232	155		22	49	54	29	
Person-years	2950	12,218	9597	4670		2950	12,218	9597	4670	
Age- and sex-adjusted	1.00 (ref)	0.78 (0.62–0.98)	0.73 (0.57–0.93)	0.86 (0.66–1.12)	0.51	1.00 (ref)	0.54 (0.33–0.89)	0.74 (0.45–1.22)	0.74 (0.42–1.29)	0.87
Multivariable model^b^	1.00 (ref)	0.82 (0.64–1.04)	0.79 (0.61–1.01)	0.90 (0.69–1.18)	0.74	1.00 (ref)	0.53 (0.32–0.90)	0.75 (0.45–1.27)	0.77 (0.43–1.40)	0.71
**Herring/mackerel** servings (median)	**<1/month** (0/week)	**1–3/month** (0.5/week)	**≥1/week** (1.5/week)			**<1/month** (0/week)	**1–3/month** (0.5/week)	**≥1/week** (1.5/week)		
No. of cases	156	382	233			27	86	41		
Person-years	5616	16,453	7365			5616	16,453	7365		
Age- and sex-adjusted	1.00 (ref)	0.91 (0.75–1.10)	1.00 (0.81–1.22)		0.66	1.00 (ref)	1.14 (0.74–1.76)	1.05 (0.65–1.72)		0.97
Multivariable model^b^	1.00 (ref)	0.99 (0.82–1.21)	1.02 (0.82–1.27)		0.79	1.00 (ref)	1.28 (0.81–2.02)	1.00 (0.59–1.67)		0.58
**Salmon/whitefish/char** servings (median)	**<1/month** (0/week)	**1–3/month** (0.5/week)	**≥1/week** (1.5/week)			**<1/month** (0/week)	**1–3/month** (0.5/week)	**≥1/week** (1.5/week)		
No. of cases	371	323	77			76	65	13		
Person-years	12,283	14,375	2777			12,283	14,375	2777		
Age- and sex-adjusted	1.00 (ref)	0.92 (0.79–1.07)	0.91 (0.71–1.17)		0.37	1.00 (ref)	0.87 (0.63–1.22)	0.78 (0.43–1.41)		0.34
Multivariable model^b^	1.00 (ref)	0.99 (0.84–1.17)	0.91 (0.70–1.18)		0.49	1.00 (ref)	0.89 (0.62–1.29)	0.80 (0.43–1.48)		0.42
**Cod/saithe/fish fingers** servings (median)	**<1/month** (0/week)	**1–3/month** (0.5/week)	**≥1/week** (1.5/week)			**<1/month** (0/week)	**1–3/month** (0.5/week)	**≥1/week** (1.5/week)		
No. of cases	96	319	356			24	55	75		
Person-years	2921	14,416	12 097			2921	14,416	12,097		
Age- and sex-adjusted	1.00 (ref)	0.70 (0.55–0.88)	0.82 (0.65–1.03)		0.61	1.00 (ref)	0.48 (0.29–0.77)	0.73 (0.46–1.15)		0.42
Multivariable model^b^	1.00 (ref)	0.71 (0.56–0.90)	0.86 (0.68–1.09)		0.41	1.00 (ref)	0.43 (0.26–0.71)	0.74 (0.45–1.20)		0.24
**Shellfish** servings (median)	**<1/month** (0/week)	**1–3/month** (0.5/week)	**≥1/week** (1.5/week)			**<1/month** (0/week)	**1–3/month** (0.5/week)	**≥1/week** (1.5/week)		
No. of cases	336	346	89			69	70	15		
Person-years	9752	16,426	3257			9752	16,426	3257		
Age- and sex-adjusted	1.00 (ref)	0.86 (0.74–1.00)	1.00 (0.79–1.27)		0.75	1.00 (ref)	0.81 (0.58–1.13)	0.80 (0.46–1.39)		0.33
Multivariable model^b^	1.00 (ref)	0.91 (0.77–1.08)	1.00 (0.78–1.29)		0.95	1.00 (ref)	0.90 (0.62–1.31)	0.98 (0.54–1.79)		0.89

a Total fish is the sum of herring/mackerel, salmon/whitefish/char and cod/saithe/fish fingers.

b Adjusted for attained age, sex, time since diabetes diagnosis (years, continuous), BMI (kg/m^2^; <20, 20–24.9, 25–29.9, ≥30) , physical activity (<20, 20–40, >40 min of walking or bicycling per day), education (primary school, high school, university), cigarette smoking (never, former <10 pack-years or ≥10 pack-years, current <20 pack-years or ≥20 pack-years), total energy intake (kcal/day; sex-specific quartiles), alcohol (g/day, sex-specific quartiles), history of high cholesterol (yes/no), history of hypertension (yes/no) and DASH diet component score (based on intake of fruits, vegetables, nuts and legumes, low-fat dairy, whole grains, sodium, sweetened beverages, and red and processed meats; quartiles). Individual fish and shellfish items were mutually adjusted.

Additional adjustment for family history of MI before age of 60 years (yes/no), aspirin use (yes/no) or fish oil supplement use (yes/no) did not change any of the associations; these covariates were therefore not included in the final models. Excluding the first 2 years of follow-up (MI: 49 cases; stroke: 34 cases; total mortality: 29 cases; CHD-related mortality: 8 cases) had no marked impact on the observed associations (>3 servings/week *vs.* ≤3 servings/month of total fish, MI: HR, 0.56; 95% CI, 0.35–0.88; stroke: HR, 1.11; 95% CI, 0.68–1.81; total mortality: HR, 0.87; 95% CI, 0.66–1.16; CHD-related mortality: HR, 0.71; 95% CI 0.38–1.31).

In analyses stratified by diabetes duration (less or more than 6 years), the inverse HRs of MI and mortality outcomes observed at the lowest consumption level (1–<2 servings/week of total fish) were stronger among those with shorter duration, whereas less marked differences were seen at higher consumption levels (Supplementary Table 1). However, no significant interactions by diabetes duration were detected (p > 0.11).

We further tested for interactions with age using alternative analyses with follow-up time as the time scale. In the analysis of MI, there was a statistically significant interaction between total fish consumption and age (p = 0.04). To explore this further, we performed a stratified analysis by age (below or above the median age of 65 years), and found that the strong inverse association was restricted to the older age group (<3 servings/week *vs.* ≤3 servings/month: HR, 0.99; 95% CI, 0.50–1.99 and HR, 0.42; 95% CI, 0.24–0.73 for <65 years and ≥65 years, respectively). No interactions were detected between total fish consumption and age for the outcomes stroke (p = 0.82), total mortality (p = 0.15) or CHD-related mortality (p = 0.38).

We also assessed fried fish consumption (highest *vs.* lowest tertile; ≥5 *vs.* ≤2 servings/month) in relation to MI, stroke and mortality. The associations were largely similar as for total fish consumption, with multivariable HRs of 0.69 (95% CI, 0.51–0.93) for MI, 1.25 (95% CI, 0.93–1.67) for stroke, 0.91 (95% CI, 0.75–1.10) for total mortality and 0.74 (95% CI, 0.47–1.15) for CHD-related mortality.

## 4 Discussion

Among individuals with diagnosed type 2 diabetes at baseline, fish consumption was associated with lower incidence of MI but not with stroke. There was also an indication that fish consumption may be inversely associated with mortality, particularly with CHD-related deaths. Stratified analyses suggested some differences in results by age and duration of type 2 diabetes. The inverse association between total fish consumption and MI was restricted to the younger half of the study population (<65 years). For MI as well as for the mortality outcomes, the risk reduction already at low consumption levels of 1–<2 servings/week was observed only among participants who had had type 2 diabetes less than 6 years, potentially indicating less severe/non-insulin treated disease. Whether results from these subgroup analyses reflect true differences or chance findings remains to be examined in future studies.

Numerous observational studies have assessed the association between fish consumption and CVD [1], [2], [3] and mortality [4]. However, few previous studies have evaluated these associations specifically in populations with diabetes [10], [11], [12], [13], [14]. Findings from the present study are in line with two previous prospective studies. In the Nurses' Health Study, both incident CHD events (326 cases) and total mortality (468 cases) were evaluated among 5103 women with type 2 diabetes. Fish consumption was inversely associated with both endpoints in a dose–response manner. The inverse associations were suggested already at very modest intakes of 1–3 servings/month compared with less than 1 serving/month (HR, 0.70; 95% CI, 0.48–1.03 for CHD incidence and HR, 0.75; 95% CI, 0.53–1.07 for total mortality) [10]. In our data, using less than 1 serving/month as the reference for total fish consumption was not possible due to a limited number of participants in this consumption category (n = 45). In a publication from the Southern Community Cohort Study, a modest statistically non-significant inverse association between total fish consumption and total mortality was observed among 16,427 participants with diabetes at baseline (HR, 0.89; 95% CI, 0.77–1.03 for highest *vs.* lowest quintile) [13]. Three other smaller studies showed no associations between total fish consumption and CHD (661 participants with diabetes/117 CHD cases) [11], MI (317 participants with diabetes/65 MI cases) [12], or total mortality (1013 participants with diabetes/80 deaths) [14]. To the best of our knowledge, no studies have previously evaluated the association between fish consumption and stroke among participants with type 2 diabetes.

Benefits of fish consumption in relation to CVD have to a large extent been attributed to the content of long-chain omega-3 fatty acids, which may reduce risk through anti-arrhythmic, anti-inflammatory, anti-thrombotic, as well as triglyceride-lowering, blood pressure-lowering and heart rate-lowering mechanisms [24]. Other factors in fish also deserve consideration. First, there is growing support for a cardioprotective role of vitamin D [25], for which fish is one of the main dietary sources [26]. Second, fish is a unique source of protein given the low content of saturated fat compared with other animal sources. Further, the amino acid profile differs from other protein sources and specific amino acids high in fish, like taurine, arginine and glutamine, may modify CVD risk [27], [28]. Fish protein has also been shown to improve insulin sensitivity in insulin-resistant individuals [29]. In addition, fish is an important source of B vitamins and trace elements like selenium and iodine. It cannot be excluded, however, that inverse associations between fish consumption and CVD and mortality in part could be explained by an overall health-consciousness associated with fish consumption. On the negative side, there are also potential adverse effects of contaminants present in certain types of fish, such as persistent organic pollutants and methyl mercury [30], [31], [32]. In the SMC, dietary exposure to polychlorinated biphenyls (PCBs) has been associated with higher risk of both MI and stroke [33], [34]. However, the accumulated evidence indicates that potential risks of fish consumption are exceeded by the benefits [35].

Strengths of this study include its prospective design, virtually complete follow-up for CVD and mortality through national registers, and detailed information on diet and other lifestyle characteristics. Further, the identification of participants with baseline type 2 diabetes did not rely on self-report. There are also some limitations of this study. First, because of the observational study design we cannot exclude that residual or unmeasured confounding may have affected our results. Second, the reliance on self-reported information of diet and other lifestyle characteristics at a single time point inevitably leads to some degree of exposure misclassification. However, misclassification would be non-differential because of the prospective design, and therefore most likely have attenuated the association. Moreover, we did not have information about diabetes medication at baseline, as the Swedish Prescribed Drug Register started in July 2005. Further, we had no information on glycemic control or presence of other diabetic complications, and although we adjusted for self-reported history of high cholesterol and hypertension, we did not have information on treatment or severity of these cardiovascular risk factors. Lastly, as our study population consisted of middle-aged and elderly Swedish women and men (predominantly white), the generalizability to younger populations and populations with other ethnicities may be limited. Further, because of incomplete registry coverage at the time of the baseline examination, we have inevitably missed to include some participants with pre-baseline type 2 diabetes. The study population can thus not be considered to be fully representative of the entire type 2 diabetes population. The incomplete inclusion is however unlikely to be related to fish consumption habits, and should therefore not have introduced selection bias.

In conclusion, fish consumption was associated with lower incidence of MI among women and men with type 2 diabetes, whereas no association was observed with incidence of stroke. Our data further indicated an association of fish consumption with lower mortality, particularly for CHD-related deaths. These findings support the current general advice on regular fish consumption also in the high risk group of type 2 diabetes patients.

## Conflict of interest

The authors declare that there is no conflict of interest.
