# Supplementary material for: Fish consumption in relation to myocardial infarction, stroke and mortality among women and men with type 2 diabetes: A prospective cohort study
Source: Clin Nutr. 2018 Apr;37(2):590–6. doi: 10.1016/j.clnu.2017.01.012 (PMC5851676; doi:10.1016/j.clnu.2017.01.012)
Supplement: Supplementary file 1 [file mmc1.docx]

**Supplementary Table 1.** Hazard ratios of myocardial infarction, stroke, total and coronary heart disease-related mortality according to categories of total fish in 2225 women and men with type 2 diabetes, the Cohort of Swedish Men and the Swedish Mammography Cohort 1998-2012; stratified by diabetes duration (less or more than 6 years)

|  | Total fish consumption servings (median) | | | | **P_trend_** |
| --- | --- | --- | --- | --- | --- |
|  | ≤3 /month  (0.5 /week) | 1- <2 /week  (1.4 /week) | 2-3 /week  (2.4 /week) | **>3 /week**  (3.5 /week) |  |
| **MYOCARDIAL INFARCTION** |  |  |  |  |  |
| *Diabetes duration <6 years* |  |  |  |  |  |
| No. of cases | 26 | 62 | 58 | 25 |  |
| Person-years | 1322 | 6349 | 4823 | 2280 |  |
| Age- and sex-adjusted | 1.00 (ref) | 0.49 (0.31-0.78) | 0.61 (0.38-0.97) | 0.51 (0.29-0.89) | 0.23 |
| Multivariable model^1^ | 1.00 (ref) | 0.54 (0.34-0.87) | 0.65 (0.40-1.07) | 0.61 (0.34-1.09) | 0.47 |
| *Diabetes duration ≥6 years* |  |  |  |  |  |
| No. of cases | 22 | 68 | 49 | 23 |  |
| Person-years | 1247 | 4565 | 3725 | 1878 |  |
| Age- and sex-adjusted | 1.00 (ref) | 0.82 (0.51-1.33) | 0.69 (0.42-1.14) | 0.64 (0.35-1.15) | 0.09 |
| Multivariable model^1^ | 1.00 (ref) | 0.81 (0.49-1.33) | 0.64 (0.37-1.09) | 0.60 (0.32-1.12) | 0.06 |
| **STROKE** |  |  |  |  |  |
| *Diabetes duration <6 years* |  |  |  |  |  |
| No. of cases | 14 | 67 | 55 | 28 |  |
| Person-years | 1322 | 6349 | 4823 | 2280 |  |
| Age- and sex-adjusted | 1.00 (ref) | 0.98 (0.54-1.72) | 1.09 (0.61-1.97) | 1.01 (0.53-1.92) | 0.75 |
| Multivariable model^1^ | 1.00 (ref) | 0.92 (0.51-1.67) | 1.03 (0.56-1.90) | 0.90 (0.46-1.78) | 0.99 |
| *Diabetes duration ≥6 years* |  |  |  |  |  |
| No. of cases | 17 | 68 | 39 | 33 |  |
| Person-years | 1247 | 4564 | 3725 | 1878 |  |
| Age- and sex-adjusted | 1.00 (ref) | 1.05 (0.61-1.78) | 0.68 (0.39-1.21) | 1.10 (0.61-1.98) | 0.67 |
| Multivariable model^2^ | 1.00 (ref) | 1.03 (0.60-1.79) | 0.67 (0.37-1.22) | 1.09 (0.58-2.04) | 0.65 |
| **TOTAL MORTALITY** |  |  |  |  |  |
| *Diabetes duration <6 years* |  |  |  |  |  |
| No. of cases | 47 | 137 | 115 | 75 |  |
| Person-years | 1506 | 7022 | 5390 | 2471 |  |
| Age- and sex-adjusted | 1.00 (ref) | 0.63 (0.45-0.88) | 0.70 (0.50-0.98) | 0.79 (0.55-1.15) | 0.99 |
| Multivariable model^1^ | 1.00 (ref) | 0.65 (0.46-0.92) | 0.75 (0.52-1.07) | 0.92 (0.62-1.36) | 0.42 |
| *Diabetes duration ≥6 years* |  |  |  |  |  |
| No. of cases | 45 | 155 | 117 | 80 |  |
| Person-years | 1444 | 5196 | 4207 | 2199 |  |
| Age- and sex-adjusted | 1.00 (ref) | 0.99 (0.71-1.39) | 0.78 (0.55-1.10) | 0.95 (0.66-1.37) | 0.33 |
| Multivariable model^1^ | 1.00 (ref) | 0.96 (0.68-1.36) | 0.84 (0.58-1.21) | 1.02 (0.69-1.51) | 0.90 |
| **CHD-RELATED MORTALITY** |  |  |  |  |  |
| *Diabetes duration <6 years* |  |  |  |  |  |
| No. of cases | 12 | 22 | 24 | 14 |  |
| Person-years | 1507 | 7022 | 5390 | 2471 |  |
| Age- and sex-adjusted | 1.00 (ref) | 0.39 (0.19-0.79) | 0.60 (0.30-1.20) | 0.66 (0.30-1.43) | 0.88 |
| Multivariable model^1^ | 1.00 (ref) | 0.35 (0.17-0.74) | 0.59 (0.28-1.24) | 0.77 (0.33-1.80) | 0.52 |
| *Diabetes duration ≥6 years* |  |  |  |  |  |
| No. of cases | 10 | 27 | 30 | 15 |  |
| Person-years | 1444 | 5196 | 4207 | 2199 |  |
| Age- and sex-adjusted | 1.00 (ref) | 0.79 (0.38-1.65) | 0.94 (0.46-1.93) | 0.89 (0.40-1.99) | 0.92 |
| Multivariable model^1^ | 1.00 (ref) | 0.88 (0.40-1.90) | 1.12 (0.51-2.46) | 1.13 (0.47-2.52) | 0.49 |

^1^ Adjusted for attained age, sex, time since diabetes diagnosis (years, continuous), BMI (kg/m^2^; <20, 20-24.9, 25-29.9,≥30) , physical activity (<20, 20-40, >40 minutes of walking or bicycling per day), education (primary school, high school, university), cigarette smoking (never, former <10 pack-years or ≥10 pack-years, current <20 pack-years or ≥20 pack-years), total energy intake (kcal/day; sex-specific quartiles), alcohol (g/day, sex-specific quartiles), history of high cholesterol (yes/no), history of hypertension (yes/no) and DASH diet component score (based on intake of fruits, vegetables, nuts and legumes, low-fat dairy, whole grains, sodium, sweetened beverages, and red and processed meats; quartiles).
